# Malaria prevention interventions beyond long-lasting insecticidal nets and indoor residual spraying in low- and middle-income countries: a scoping review

**DOI:** 10.1186/s12936-022-04052-6

**Published:** 2022-02-02

**Authors:** Sarah Nalinya, David Musoke, Kevin Deane

**Affiliations:** 1 Department of Disease Control and Environmental Health, School of Public Health, College of Health Sciences, Makerere University, Kampala, Uganda; 2 Open University, Milton Keynes, UK

**Keywords:** Malaria, Housing design, Preventive interventions, Integrated vector management, Repellents, Mosquitoes, LMICs

## Abstract

**Background:**

Significant progress in malaria prevention during the past two decades has prompted increasing global dialogue on malaria elimination. Recent reviews on malaria strategies have focused mainly on long-lasting insecticidal nets (LLINs) and indoor residual spraying (IRS), with little emphasis on other prevention methods. This article is a scoping review of literature on malaria prevention methods beyond LLINs and IRS in low- and middle-income countries (LMICs).

**Methods:**

This scoping review found articles published between from 1994 to 2020. Studies were obtained from a search of the PubMed, the Cochrane Library and Social Science abstracts. Grey literature and manual search of secondary references was also done. The search strategy included all study designs but limited only to English. Three independent reviewers performed the selection and characterization of articles, and the data collected were synthesized qualitatively.

**Results:**

A total of 10,112 studies were identified among which 31 met the inclusion criteria. The results were grouped by the 3 emerging themes of: housing design; mosquito repellents; and integrated vector control. Housing design strategies included closing eves, screening of houses including windows, doors and ceilings, while mosquito repellents were mainly spatial repellents, use of repellent plants, and use of plant-based oils. Integrated vector control included larvae source management. Evidence consistently shows that improving housing design reduced mosquito entry and malaria prevalence. Spatial repellents also showed promising results in field experiments, while evidence on repellent plants is limited and still emerging. Recent literature shows that IVM has been largely ignored in recent years in many LMICs. Some malaria prevention methods such as spatial repellents and IVM are shown to have the potential to target both indoor and outdoor transmission of malaria, which are both important aspects to consider to achieve malaria elimination in LMICs.

**Conclusion:**

The scoping review shows that other malaria prevention strategies beyond LLINs and IRS have increasingly become important in LMICs. These methods have a significant role in contributing to malaria elimination in endemic countries if they are adequately promoted alongside other conventional approaches.

**Supplementary Information:**

The online version contains supplementary material available at 10.1186/s12936-022-04052-6.

## Background

Malaria continues to be a significant global health issue, with an estimated 229 million malaria cases and 409,000 deaths in 2019 in 85 endemic countries, a reduction from 238 million cases and 736,000 deaths in 2000 [1]. Whilst there has been recent progress in the reduction of malaria morbidity and mortality, the last 5 years have seen a limited reduction in the incidence of the disease. Indeed, the global malaria incidence in 2018 was nearly the same as in 2014, with slowing of improvements in the malaria mortality rate [2], and increases in cases between 2015 and 2017 in 55 countries [3]. This stalling and in some cases reversal of progress, emphasizes the need for a renewed focus on controlling malaria if the vision of an ‘Africa Free of Malaria’ [4] is to be realized, and if the disease is to be eradicated across the globe.

Central to malaria prevention efforts are the use of long-lasting insecticidal nets (LLINs) and indoor residual spraying (IRS), alongside improved treatment regimens and expanded testing programmes [3]. The efficacy of these approaches is well established, and they are viewed as fundamental to malaria eradication. However, in the World Health Organization (WHO)-African region, which accounts for 93% of all malaria cases, coverage of these methods currently remains below national and international targets [5, 6]. There are a wide range of well-documented barriers to uptake of LLINs and IRS including: a lack of availability [5, 7, 8]; economic costs where free distribution programmes are absent [5, 9, 10]; a lack of education or knowledge about the protective effect of LLINs [5, 7, 11–14]; low seasonal use of LLINs during the dry season or other times of the year where it is believed malaria risk is low [5]; concerns regarding discomfort of LLINs due to lack of airflow [5, 13], heat and skin irritation [13]; problems associated with hanging nets up or having the space to do so [5, 10]; challenges related to use of LLINs by those who sleep outdoors [9]; alternative uses for insecticide-treated nets (ITNs) including for protecting seedlings, as fishing nets and curtains [10, 11, 15]; concerns around ineffectiveness of IRS [14, 16]; negative experiences of previous spraying [16]; residual effects of spraying [16]; and the need to remove household goods for spraying [16]. Whilst there is no doubt that when used appropriately, LLINs and IRS have a significant impact on the incidence of malaria, the stalling of progress noted above raises questions concerning the direction of malaria prevention efforts, and particularly with respect to whether policy makers should focus more attention on alternative or complementary interventions.

Evidence from across Africa suggests that the success of LLINs and IRS in targeting species that primarily rest indoors has led to a change in patterns of transmission, with species that are more flexible in feeding and resting behaviours now more prominent vectors. For example, *Anopheles gambiae *sensu stricto (*s.s*.) has been historically considered the major malaria vector in Africa [17–20]. However, the widespread use of LLINs and IRS has led to a significant decline of this species in many areas, shifting the majority of transmission to *Anopheles arabiensis* [21–24]. The result is an increase in transmission from outdoor biting, a mode of transmission that IRS and ITN do not target. In addition, key malaria vectors have increasingly become resistant to the insecticide used to treat bed nets and in IRS [25–27], presenting a further challenge to a malaria reduction strategy that is highly reliant on these two methods. Another concern is that despite the clear correlation between high malaria rates and low socio-economic status, with malaria disproportionately impacting the poor, it is not clear that the reasons for this are fully understood and incorporated into policy making and intervention design. In the recent Lancet commission, the focus on the role of poverty and interventions to address poverty-related risk was underwhelming [28], with attention primarily focused on the need for increased spending, political commitment and improved programme management [3]. Indeed, a recent review concluded that “no progress has been made in the analysis of social categories—territory, social class, gender, ethnic group, macroeconomic policies—or other socioeconomic characteristics that determine risk of illness or death from malaria” [29], emphasizing potential limitations in the way that malaria transmission is understood and thus the development of interventions that can potentially address distal factors.

The irregular uptake of the core malaria prevention interventions, combined with uneven progress, changing dynamics of vector transmission, and lack of focus on the social determinants of malaria transmission, casts doubt on whether increased funding and focus on the further rollout of LLINs and IRS, that is ‘more of the same’, will be sufficient to meet international goals regarding eradication, and highlights the importance of considering alternative interventions beyond these two methods. This scoping review, therefore, aimed at documenting the range of alternative interventions that have been implemented in the field to reduce malaria transmission, and to summarise the current state of evidence regarding the efficacy of these interventions, potential barriers, and the suitability of these interventions for widespread implementation in low- and middle-income countries (LMICs). The review also provides national, regional and global stakeholders with an expanded literature on promising interventions where more evidence is required, and an assessment of the extent to which the social determinants of malaria are addressed by these alternative interventions.

## Methods

### Search strategy

This literature search followed a scoping review methodology because there is limited published literature that evaluates malaria prevention methods that do not involve the use LLINs and IRS. Therefore, two key research questions guided the literature search:

1. What interventions that do not involve the use of LLINs and IRS have been used to reduce malaria transmission in LMICs?
2. How are these interventions are linked to the social determinants of malaria in those communities?

Published articles were searched through different databases such as PubMed, Cochrane Library, Sociological Abstracts and Google Scholar (Additional file 1: Table S2). The search was limited to articles published in English. The key concepts used in the search included “novel/new approaches to malaria prevention”, “unconventional methods for malaria prevention”, “social determinants of malaria” and “low-and-middle income countries.” More articles were sought via a manual search of the references from the retrieved articles. Keywords of the key concepts were modified accordingly following the first search cycle to minimise any misunderstanding caused by their meanings when unaccompanied by the relevant key concepts. This was done to find articles on malaria prevention that do not involve LLINs and IRS. The key concepts were paired using Boolean operators. A separate search for each concept was conducted using keywords in the databases. They were (“malaria prevention”, OR “malaria control”, OR “malaria interventions”) AND “social-determinants” AND “low-and middle-income countries”. The results for each concept were paired using the “AND” operator to find articles focusing on all the key concepts. In addition, grey literature was searched aimed at finding relevant articles that were not published in commercial publication databases and targeting a limited audience [30]. This search enabled us to obtain articles from government and non-government organisations which funded or implemented malaria prevention initiatives in LMICs. Whilst, as noted above, Africa accounts for the majority of malaria cases, the literature search was expanded to include all low- and-middle-income countries (including Asia) to capture all possible interventions given that different countries and regions can learn from the experiences of others. The search produced articles which focused on the prevention of malaria while also addressing the social determinants of malaria.

### Study selection criteria

Articles were included in this scoping review following the Population, Concept, Content (PCC) format [31]. Therefore, the articles had to meet all the three inclusion criteria below:

1. Population: They were conducted in a low- and middle- income country (LMIC).
2. Concept: They focused on malaria prevention interventions other than LLINs or IRS and considered the wider social determinants of malaria. These interventions included vector control, environmental management and personal protection.
3. Content: Published and unpublished articles including clinical trials, case–control studies, cross-sectional studies, and other epidemiological studies were included in this review. The interventions in the studies that were considered had that the following outcomes: they all had an effect or impact on the malaria burden in the community or field site, benefited the participants/community through improving their knowledge, attitude and practices of malaria prevention; led to reduction of malaria transmission in the community through directly or indirectly addressing the social determinants of malaria; and were affordable hence had the potential to be sustainable in low income settings. Affordability of an intervention was defined both as a measure of the available resources the intervention consumes, and also as a share of the resources left after spending on essential goods such as food [32]. An assessment of how the required resources could have influenced the use of a given malaria prevention intervention at household level was conducted. Studies with interventions that were assessed to be affordable using both measures were included in the scoping review.
4. Articles meeting the above criteria were included regardless of their publication year, methodology or scope. Articles which focused on interventions which were not primarily aimed at malaria prevention were excluded.

### Data extraction and synthesis

Data extraction was conducted in five steps. First, the retrieved articles were screened using their titles and abstracts. The selected articles were further screened by reading full texts, then those that fully met the inclusion criteria were selected to be included in the review. Data from each included article was recorded on a data extraction form (Additional file 1: Table S3). The recorded data included the first author, year of publication, area of study, methodology, study participants, intervention of focus, and major results. The synthesis of quantitative results consisted of extracting results and recommendations from similar studies and comparing them, depending on the context in which they were implemented. The data from qualitative studies was categorised and coded using Atlas.ti 8 [33] and a coding framework was developed for this study (Additional file 1: Table S4). The resulting codes and their meaning units were thematically analysed to obtain emerging themes. All relevant results of were summarized in tabular form under the themes that emerged.

### Methodological quality appraisal

Since the objective of this review was to scope and map existing literature on malaria prevention methods beyond LLINs and IRS, methodological quality or bias risk of the included articles was not assessed. This is consistent with guidance on conducting scoping reviews [34].

### Replicability of the search

Although a rigorous plan was followed while conducting a comprehensive search for articles to include in this scoping review, claims of replicability of the results cannot be made. Although future studies may be able to replicate the search methods used in this review, it is very likely that their results would be different, which is common when searching electronic databases [30].

## Results

**Fig. 1:**
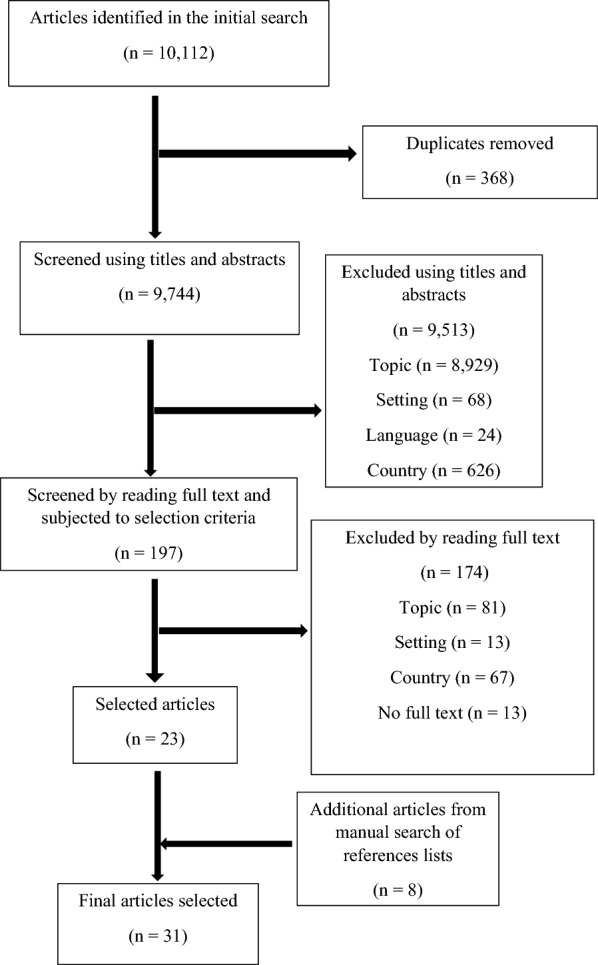
PRISMA flow diagram

The initial search resulted in a total of 10,112 articles. After eliminating duplicates, the remaining 9744 articles were screened using their titles and abstracts among which 197 articles qualified and were further screened by reading the full articles. These articles were then subjected to the PCC inclusion criteria of which 23 were selected to be included in this scoping review. A manual search from the reference list of the selected articles resulted to an additional 8 articles hence 31 articles were reviewed (Fig. 1). Characteristics of the included studies are in Table 1.

**Table 1:** Characteristics of the included studies

No	Study	Publication year	Study design	Country	Intervention studied
1	Lindsay et al	2003	Randomised control trial	Gambia	Ceilings
2	Ogoma et al	2010	Field experiment	Tanzania	House screening and ceilings
3	Mburu et al	2018	Field experiment	Malawi	Closing eaves
4	Mmbando et al	2018	Field experiment	Tanzania	Modification of eaves (transfluthrin-treated eave ribbons)
5	Swai et al	2019	Field experiment	Tanzania	Modification of eaves (transfluthrin-treated eave ribbons)
6	Snetselaar et al	2017	Semi-field experiment	Kenya	Modification of eaves (insecticide-treated eave tubes)
7	Mwanga et al	2019	Semi-field experiment	Tanzania	Modification of eaves (transfluthrin-treated eave ribbons)
8	Sternberg et al	2016	Semi-field experiment	Tanzania	Modification of eaves (eave tubes)
9	Kirby et al	2009	Randomised control trial	Gambia	House screening
10	Bradley et al	2013	Literature review	Equatorial Guinea	Window screening and closed eaves
11	Kaindoa et al	2018	Entomological survey	Tanzania	Screening windows, closing eaves and gaps on doors
12	Kirby et al	2008	Field experiment	Gambia	Open eaves
13	Atieli et al	2009	Randomised control trial	Kenya	Ceilings
14	Ernst et al	2009	Case–control study	Kenya	Housing modification including ceilings
15	Kirkby et al	2010	Cross-sectional	Gambia	House screening and ceilings
16	Ogoma et al	2009	Cross-sectional	Tanzania	Window screening, ceilings and closed eaves
17	Kawada et al	2005	Field experiment	Indonesia	Spatial repellents
18	Kawada et al	2004	Field experiment	Indonesia	Spatial repellents
19	Kawada et al	2008	Randomised control trial	Tanzania	Spatial repellents
20	Ogoma et al	2017	Field experiment	Tanzania	Spatial repellents
21	Masalu et al	2017	Field experiment	Tanzania	Spatial repellents
22	Masalu et al	2020	Field experiment	Tanzania	Spatial repellents
23	Sambali et al	2011	Field experiment	Tanzania	Repellent plants
24	Seyoum et al	2003	Field experiment	Kenya	Repellent plants
25	Moore et al	2007	Randomised control trial	Bolivian Amazon	Essential oils
26	Tesfahuneygn and Gebreegziabher	2019	Review article	Ethiopia	Essential oils
27	Ault	1994	Observational	Mexico and Dominican Republic	Environmental management
28	Yohannes et al	2005	Clinical survey, entomology survey and action research	Ethiopia	Environmental management
29	Yasuoka et al	2006	Observational	Sri Lanka	Environmental management
30	Okech et al	2008	Cross-sectional survey	Kenya	Environmental management
31	Keiser et al	2005	Systematic review	Multiple LMICs	Environmental management

### Findings

The findings of this review present evidence on three categories of malaria prevention strategies: (1) Housing design focused on modification of eaves, ceilings and house screening; (2) Mosquito repellents related to spatial repellents, repellent plants and essential oils; and (30 Integrated vector control specifically larval source management and environmental management.

### Improving of housing design

Since the majority of malaria transmission in LMICs occurs predominantly indoors [35, 36], housing condition is an important risk factor for malaria transmission. Three studies [37–39] conducted in different malaria-endemic settings showed that improvement in the general housing design such as closing eaves and house screening results in reduction of indoor mosquito densities hence significant reductions in malaria transmission. This section presents on evidence on three aspects of housing design: modification of eaves; house screening; and ceilings.

#### Modification of eaves

Seven studies from four African countries were identified that focused on how the modification of eaves can reduce the rate of malaria transmission [40–46]. Although there was a variation of the materials used to cover the eaves, all studies showed that partially or fully closing eaves reduced mosquito entry, hence indoor biting, by 4–12 times. All the experimental studies showed that modification of eaves reduced indoor biting rates by 77% to ≥ 99%. Four of the studies [42, 44–46] showed that closing eaves with insecticide-treated inserts had the potential to protect neighbouring households which did not use the technology, hence reducing the need for individual or household compliance. Although eave modification alone may not be sufficient for malaria elimination, there is promising evidence supporting the intervention as a low-cost method that can be integrated in malaria prevention programmes especially in rural or resource-restricted communities [47].

#### House screening

Screening windows and doors is another house improvement method for which strong evidence exists as an effecting malaria prevention method. This method has been successfully used for many years to reduce mosquito bites hence malaria infections in various settings such as United States, Greece and Italy [48]. More recent studies in Africa have shown that full screening of windows and doors alone significantly reduced indoor mosquito densities; and provided valuable protection against malaria transmission in rural communities [49–51]. The strength of house screening is that it is a relatively cheap malaria prevention method since most screens usually last for a substantial period before they would require repair or replacement. Screening also does not depend as much on individual behaviour as interventions, such as LLINs.

#### Ceilings

In this review, a ceiling is defined as any overhead interior finished surface concealing the underside of the roof structure or the floor of a storey above. It can be made of various materials such as concrete, wood or papyrus. Presence of ceilings in houses has demonstrated to provide significant protection against mosquito entry, hence reduced indoor mosquito densities in sub-Saharan Africa [51, 52]. It is important to consider this modification because less than half of houses in a typical urban area in a LMIC have ceilings [53], implying an even lower proportion in rural areas. Four studies conducted in East and West Africa showed that ceilings reduced mosquito entry, and this led to reduced risk of acquiring malaria [40, 53–55]. The strength of ceilings is that it is associated with other desirable properties, such as cooler indoor temperatures and modern aesthetics [56, 57]. This increases acceptability and willingness to pay despite the high installation costs [40]. In addition, using locally available materials such as papyrus and plywood to make ceilings as showed in [40, 54] can greatly reduce the initial installation cost hence making it a relatively inexpensive method to be used alongside other malaria prevention measures.

### Mosquito repellents

Although the majority of malaria transmission in LMICs occurs indoors, the increasing importance of outdoor transmission cannot be ignored. In order for malaria to be successfully eliminated from LMICs, both indoor and outdoor transmission needs to be addressed. Mosquito repellents are one of the novel strategies for malaria prevention that have the potential to target both indoor and outdoor malaria transmission. The following section presents and examines existing literature on mosquito repellents specifically spatial repellents, repellent plants and essential oils.

#### Spatial repellents

Spatial repellents for mosquito control are pyrethroids possessing sub-lethal properties such as repulsion, deterrence, feeding inhibition and reduction of fertility. Although these repellents may not kill the vectors, they decrease their malaria-transmission capacity. Spatial repellents are distinguished from LLINs and IRS insecticide formulations by having a lower dosage and their ability to vaporise at ambient temperatures [58]. Unlike LLINs and IRS, spatial repellents do not require surface contact with the vectors and can have an effect over large areas [59]. Spatial repellents are mainly incorporated in devices such as mosquito coils, liquid vaporizers, vaporizer mats and ambient emanators [60]. The use of spatial repellents as a vector control strategy has been supported due to its various advantages. First, spatial repellents have been found to protect people against both indoor and outdoor biting [61, 62] and thus can be used to target outdoor transmission where LLINs and IRS may not be applicable. In addition, when used consistently and appropriately, spatial repellents do not require high levels of personal compliance and they protect both users and non-users [61]. The recent development of improved active ingredients in spatial repellents such as metofluthrin has increased the value of this strategy [63].

Evidence regarding the effectiveness and feasibility of spatial repellents originates from five studies conducted in Africa and Asia. Experimental studies conducted in Indonesia [64] and Tanzania [63, 65–67] showed various rates of reduced indoor biting ranging from 89 to 100% when spatial repellents were used. The studies also recorded reductions in outdoor biting from 90 to 66%. The different studies incorporated spatial repellents in different devices such as locally-made decorative baskets, emanators, plastic strips, *hessian* strips and locally-made chairs. Studies [66, 67] showed that spatial repellents were readily acceptable in the community and using local materials made them easy to scale as supplementary vector control interventions. The limitations associated with LLINs and IRS as vector control methods [23] have increased the attention towards use of repellents as a potential strategy to bridge the existing gaps [68]. Indeed, evidence has shown how spatial repellents can be creatively applied to low-cost home essentials commonly used in LMICs.

#### Repellent plants

Repellent plants are an economical method of repelling mosquitoes especially suitable for rural areas which may not afford commercial repellents or modern house screening methods. Some plants such as wild sage, lantana (*Lantana camara*); neem (*Azadirachta indica*); lemongrass (*Cymbopogon citratus*); and several of the Ocimum genus are used locally in many communities to repel mosquitoes [69]. The efficacy of repellent plants has been demonstrated by two studies. One study conducted in Tanzania showed that planting *Lantana camara* around homes significantly reduced indoor densities of *Anopheles gambiae* *s.s.* by 56% and *Anopheles funestus* *s.s.* by 83% [69]. Another study conducted in Kenya registered reductions in entry of of *An. gambiae* sensu lato (*s.l*.) into houses by 22.7% [70].

#### Plant-based essential oils

Essential oils from *Cymbopogon* grasses such as lemon grass have also been used as potent mosquito repellents. Studies from Asia and Central America have shown that essential oils from lemon grass can provide protection against mosquito bites by up to 95% [71]. Other plants from which repellent oils have been extracted include garlic (*Allium sativum*) and cinnamon (*Cinnamomum osmophloeum*), which have been shown to have insecticidal properties against larvae and adults of different mosquito species [72]. Although plant-based repellents are less effective than synthetic alternatives, they are more culturally acceptable in many community settings [73].

Mosquito repellents have not been formally integrated in the malaria control strategy in most countries [74]. However, evidence suggests that the different forms of mosquito repellents would be highly acceptable in communities if they were integrated into the formal integrated vector control strategy [75]. Mosquito repellents have the potential to become an important strategy for malaria vector control since outdoor malaria transmission is increasingly becoming important [76].

### Integrated vector management

Integrated vector management (IVM) involves the combination of a range of multiple interventions to control vector-borne diseases [77]. These methods include vector control measures which target adult mosquitoes, such as IRS [78], and environmental management measures, such as larval source management to eliminate the mosquito breeding sites; and personal protective measures such as window screening, LLINs and mosquito repellents [2]. Throughout history, programmes with an IVM approach have resulted in significant reductions in vector populations and malaria burden across different settings [79–81]. There is evidence that IVM can complement the existing core malaria prevention strategies (LLINs and IRS) by avoiding reliance on any single intervention to reduce the burden of malaria [82–84]. A report by Chanda and colleagues describes a highly successful IVM programme implemented in Zambia [85] which serves in various ways as an important success story in LMICs. However, progress of implementing IVM in developing countries is still slow, and some of the IVM strategies of larval source management and environmental management have been given little attention as malaria control tools.

#### Larval source management

Larval source management (LSM) involves the management of water bodies which can harbour the larval stage of mosquitoes to prevent the successful completion of their lifecycle. LSM was one of the major vector control interventions used in the USA, Canada and throughout Europe for over a century [86–88]. However, despite the extensive use and success of LSM in developed countries, it has been largely ignored as a core malaria control strategy in Africa and other LMICs over the past 50 years.

Recent studies have demonstrated that an increased global focus on LLINs and IRS has led to a significant decline in malaria vectors which rest and feed indoors in many LMICs, which has in turn increased the apparent importance of malaria vectors that rest and feed outdoors [24, 89, 90]. It is, therefore, essential to also explore interventions that target outdoor resting and biting malaria vectors if LMICs are to realise their target to eliminate malaria. In addition to addressing outdoor biting, the advantage of LSM is that it targets larvae which cannot escape from their breeding sites, and unlike adult vectors, cannot easily avoid control measures [22]. LSM could also have an important role in eradicating malaria in areas which are malaria 'hot spots', after the application of existing tools directed at indoor-feeding vectors. LSM has also been seen as more sustainable than the conventional vector control interventions because it can be implemented by local communities with no need for high recurrent costs [91–93]. This therefore develops local skills and adaptation, thus creating an opportunity for community empowerment for health control [94]. Although LSM cannot be implemented as a stand-alone intervention, it should be given more attention as an IVM tool.

#### Environmental management

Environmental management is another IVM strategy that involves seemly simple measures such as proper construction of drains, desilting drains, controlling vegetation cover, and ensuring proper solid waste and wastewater disposal. Such measures are aimed at good keeping of the environment, controlling flooding and water stagnation, thus minimizing the opportunities for breeding of vectors [95, 96]. Education of communities about environmental management is also essential to facilitate community participation in such activities. Although environmental management was highly promoted in the control of malaria in developed countries [97–100], its importance in the control of malaria in Africa significantly reduced when emphasis shifted to LLINs and IRS [101].

Evidence has shown that reducing opportunities for mosquito breeding through environmental management is followed by a significant decrease in mosquito populations in the surrounding communities [102–105]. For example, a community participatory environmental management study conducted in Tanzania which consisted of drain cleaning activities resulted in a significant reduction in malaria infections [97]. Similar results were observed in an earlier environmental management study conducted in Nepal in which clearing vegetation and draining stagnant water led to a 35% reduction in malaria cases in the intervention communities [102]. As the malaria global strategy shifts from control to elimination, the success of both recent and earlier environmental management interventions has prompted the WHO to refocus on the strategy as one of the cost-effective and sustainable tools for malaria prevention [106].

Environmental management offers practical opportunities for sustainable malaria control in LMICs not on its own, but as part of an IVM approach [96]. Political will and commitment, community participation, sufficient initial financial resources, and multi-sectoral collaboration [107] are key for successful environmental management implementation. Environmental management is argued to be cost-effective, easy for communities to implement and maintain, and has minimal negative effects on humans or the environment [108]. Therefore, participatory environmental management, if strategically included in an IVM programme along with other strategies such as LLINs, could contribute to further reduction in the malaria burden.

## Discussion

This scoping review of 31 studies focused on methods of prevention of malaria beyond LLINs and IRS in LMICs. One of the objectives of the review was to consider how these interventions take in consideration the wider social determinants of health such as income, education and social norms in preventing malaria. The methods established in the review are: improving housing design; use of repellents; and IVM. As many endemic countries shift from malaria control to elimination, it is important to examine literature on malaria prevention beyond the core methods. The review found that such alternative malaria prevention methods have the potential to supplement LLINs and IRS, if given the attention they deserve. The evidence shows that methods such as improving housing design and IVM have been shown to be cost-effective, sustainable and socially acceptable in many communities if adapted to suit local contexts. There is also need for malaria prevention interventions to consider the social determinants of health which greater influence community practices. Considering the social determinants of health ensures that interventions do not solely rely on individual behaviour and characteristics to be successful. For example, minimising mosquito breeding sites will reduce mosquito populations hence malaria transmission in entire communities regardless of individual behaviour. Unfortunately, although evidence strongly links malaria with the social determinants of health [109] specifically poverty and its associated factors, the current major malaria prevention interventions have not fully considered this relationship. This situation, therefore, necessitates governments and other stakeholders at national and global levels to further explore the socio, economic, cultural, and other determinants that are likely to influence the uptake of various malaria prevention methods in endemic countries.

Housing is one component of the relationship between malaria and poverty that has been substantially studied in LMICs [41, 43, 48]. Several studies have shown that housing improvements not only significantly contribute to the reduction of malaria prevalence but also have additional benefits, such as indoor temperature regulation and aesthetic qualities, which increases community acceptability and cost effectiveness of this strategy [38]. Fortunately, the use of locally available building materials may be a low-cost solution for resource-restricted communities. The findings of this study concur with those in recent systematic review which showed that improving the social determinants of health including housing reduces the prevalence of malaria in LMICs [110]. While studies have provided the necessary evidence for refocusing on social interventions such as housing improvements [110], most of the research on such low-cost housing improvements has been done as pilots on a small-scale [53], leaving questions of how such interventions can be efficiently scaled-up. This calls for further research and more commitment to a larger-scale field trials to determine the cost-effectiveness of house improvement in various community settings in LMICs.

Spatial repellents are an important malaria prevention strategy that was found worth exploring. This is because outdoor transmission of malaria has become increasingly important, partly due to the change in the important mosquito species and/or their feeding behaviour in response to LLINs and IRS. In addition, recent studies have emphasized the role of social determinants of health especially livelihoods, such as fishing or farming carried out at night and socio-cultural activities such as funerals which expose people to mosquitoes while outdoors [111–113]. The increasing importance of outdoor malaria transmission in many communities is gradually rendering the available and scalable malaria control interventions insufficient for malaria elimination [43]. However, the interventions which target outdoor biting are currently under-explored in many malaria endemic communities. The evidence suggests that spatial repellents are flexible and can be creatively incorporated in local and socially acceptable items such as decorative baskets and chairs. More epidemiological and entomological research is needed on what drives the risk of malaria transmission outdoors and the relative risk of the different night outdoor social and livelihood activities. The feasibility, acceptability and sustainability of the different interventions regarding outdoor transmission of malaria should be further studied, as this is a significant gap in the literature.

This review has shown that there is emerging evidence that some local plants and their essential oils repel mosquitoes. Although the evidence is still limited, the available studies have shown promise that some plants locally available in different communities have the potential to repel mosquitoes on their own, or their essential oils could be used as natural, low-cost mosquito repellents and insecticides [114]. As further research is conducted to fully establish how plants with mosquito-repellent properties can be fully exploited, the large divide between a more community-based approach and the commercial approach of pharmaceutical companies should be bridged. Although it is established that mosquito resistance to a wide range of phyto-components in the mosquito repellent plants is rare, the commercial approach normally concentrates on one or two extracts of a given plant hence risking resistance [115]. Global malaria programmes should, therefore, encourage further research and subsequent implementation of natural and low-cost mosquito repellent plants and essential oils in the appropriate communities in LMICs.

There is strong evidence to suggest that environmental management and LSM are important interventions for outdoor biting [116–118] and are critical in the elimination of malaria in many countries. In addition, once effectively implemented and monitored, these interventions have many aspects that are more sustainable than the current conventional malaria prevention interventions [119]. Similar to the other malaria prevention interventions presented in this review, environmental management and LSM do not rely on individual or daily compliance to be successful. Environmental management also engages with the social determinants of health, placing focus on improving infrastructure and enhancing land management in poorer areas, interventions which will also likely have additional health-related benefits. It also engages with the geographies of malaria and poverty, acknowledging that increased risk of malaria is related to where one lives, and that spatial and social distributions of malaria may overlap [96]. However, for a number of reasons these interventions have been under-emphasized and, therefore, underused in malaria programming for LMICs. For example, many LMICs still lack local capacity to implement and monitor such context-specific and relatively long-term environmental management interventions [120]. There is urgent need to re-incorporate these interventions as key components of the IVM strategy. The inclusion of environmental management and LSM in the Global Malaria Action Plan of the Roll Back Malaria Partnership [121] promises renewed interest in these interventions as many LMICs countries move towards malaria elimination. Programmatic implementation and assessment of locally appropriate systems for delivering these environmental management interventions that target outdoor control of mosquitoes in LMICs are therefore necessary for the further reduction of malaria in LMICs.

It is worth noting that many of the none-core methods explored in this scoping review require little or no individual behaviour change, an issue that much of the literature concerning the use of LLINs and ITNs has attempted to unpack. When considering the social determinants of health, this helps to shift focus away from stigmatising research that focuses on why poorer populations may make ‘worse’ health-related decisions. However, some of the alternative interventions noted in this review are more holistic and can be more difficult to implement, monitor and evaluate because they require bottom-up approaches to communities. As a result, these interventions may require greater political commitment, multi-sectoral collaboration and longer implementation periods. Such complexities may be the reason why interventions such as LSM and environmental management have lost the interest of governments and donors in favour of biomedical and top-down approaches that are easier to quantify, monitor and evaluate [109]. Further progress in malaria control may require malaria control programmes to devise means of incorporating these various community centred but effective strategies instead of avoiding them.

## Conclusion

This scoping review found promising evidence on improving housing design, use of mosquito repellents, and integrated vector control to contribute to malaria prevention efforts in LMICs. Furthermore, the social determinants of malaria transmission need to be addressed in order to tackle the bold vision of global eradication. These include poor housing conditions that expose communities to mosquitoes, and poverty that limits communities’ ability to take-up and sustain malaria control measures. There is also need for governments, academia and other stakeholders to strengthen malaria research to lead into sustainable interventions. As the ‘one size fits all’ approach to malaria prevention has shown to be insufficient in LMICs, country-specific and well-coordinated integrated malaria prevention approaches need to be adopted to make significant progress towards malaria elimination and subsequently global eradication.

## Supplementary Information

**Additional file 1.** Summary of databases searched, data extraction template and coding framework of the study.

## Data Availability

Not applicable.

## Abbreviations

IRS: Indoor residual spraying
ITNs: Insecticide-treated mosquito nets
IVM: Integrated vector management
LLINs: Long-lasting insecticidal nets
LMIC: Low- and middle-income country
LSM: Larval source management
WHO: World Health Organization
