# Supplementary material for: Malaria prevention interventions beyond long-lasting insecticidal nets and indoor residual spraying in low- and middle-income countries: a scoping review
Source: Malar J. 2022 Feb 2;21:31. doi: 10.1186/s12936-022-04052-6 (PMC8812253; doi:10.1186/s12936-022-04052-6)
Supplement: Supplementary file 1 — Additional file 1. Summary of databases searched, data extraction template and coding framework of the study. [file 12936_2022_4052_MOESM1_ESM.docx]

**Appendix: Summary of databases searched, data extraction template and coding framework of the study**

**Table 2: Summary of the databases searched**

| Database | Search Terms | Number of articles retrieved |
| --- | --- | --- |
| PubMed | ("malaria prevention"[MeSH Terms] OR "malaria control"[tiab] OR "malaria interventions"[tiab] AND ("social determinants"[Mesh] AND "low-and middle-income countries"[tiab]) | 6,022 |
| Malaria journal | 'malaria prevention' OR 'malaria control' OR 'malaria interventions' AND 'social determinants' AND 'low-and middle-income countries' | 328 |
| Google Scholar | (TITLE-ABS-KEY (malaria prevention) OR TITLE-ABS-KEY (malaria control) OR TITLE-ABS-KEY (malaria intervention) AND (TITLE-ABS-KEY (social determinants) AND TITLE-ABS-KEY (low- and middle-income countries) | 4,133 |
| The Lancet | ‘malaria prevention' OR 'malaria control' OR 'malaria interventions' AND 'social determinants' OR 'social determinants' AND 'low-and middle-income countries' | 71 |
| WHO website | ‘malaria prevention', 'malaria control', 'malaria interventions', 'social determinants', 'low- and middle-income countries' | 21 |
|  |  |  |

**Table 3: Data extraction template**

| **Study characteristics** | **Review inclusion criteria** | **Yes/No** |
| --- | --- | --- |
| **First author** |  |  |
| **Publication year** |  |  |
| **Language** | English |  |
| **Full text available** |  |  |
| **Type of study** | Randomised control trial  Field experiment  Observational  Case-control  Cross-sectional  Review  Other (specify) |  |
| **Participants/population** | Low- and middle-income country |  |
| **Setting (Including location and social context)** | Low- and middle-income country  Malaria endemic setting |  |
| **Study aim** | Malaria prevention  Malaria control  Malaria elimination |  |
| **Type of intervention** | Intervention that did not involve LLINs or IRS |  |
| **Methods used** | Quantitative  Qualitative  Mixed methods |  |
| **Study outcome/results** | Impact on malaria prevention and control knowledge or practices |  |
| **Decision on inclusion** |  |  |

**Table 4: Qualitative Coding framework**

| **Theme** | **Sub-theme** | **Code** |
| --- | --- | --- |
| Housing design | Eaves  Ceiling  Screening | Closed eaves, open eaves  Presence of ceiling, type of ceiling, cost of ceiling, advantages of ceiling  Screening windows, screening ventilators, types of screens |
| Repellents | Natural repellents  Synthetic repellents | Plant repellents, essential oils  Vapourisers, mosquito coils, repellent strips, emanators |
| Integrated vector management | Larval source management  Environmental management | Advantages of LSM, feasibility of LSM, success of LSM  Drainage, environmental sanitation, flooding control, waste-water disposal, success of EM |
